# Influence of Ocular Residual Astigmatism on the Correction of Myopic Astigmatism by Toric Implantable Collamer Lens: A Comparative Study With Femtosecond Laser Small Incision Lenticule Extraction

**DOI:** 10.3389/fmed.2022.828492

**Published:** 2022-06-13

**Authors:** Ling Sun, Xiaoyu Zhang, Lan Ding, Yang Shen, Yishan Qian, Xingtao Zhou

**Affiliations:** ^1^Department of Ophthalmology, Eye and ENT Hospital, Fudan University, Shanghai, China; ^2^Laboratory of Myopia, Chinese Academy of Medical Sciences, Shanghai, China; ^3^Shanghai Research Center of Ophthalmology and Optometry, Shanghai, China

**Keywords:** ocular residual astigmatism, myopic astigmatism corrections, toric implantable collamer lens, femtosecond laser small incision lenticule extraction, vector analysis

## Abstract

**Purpose:**

To evaluate the influence of the origin of astigmatism on the correction of myopic astigmatism by toric implantable collamer lens (TICL) and compare it with femtosecond laser small incision lenticule extraction (SMILE).

**Methods:**

Ocular residual astigmatism (ORA) was determined by vector analysis using manifest refraction and Scheimpflug camera imaging of the anterior cornea. One-to-one matching between the TICL and SMILE groups was performed by preoperative manifest refractive astigmatism (RA) and ORA, tolerating a maximum difference of 0.50 diopter (D) for RA and 0.25 D for ORA. Patients of each group were further divided into groups according to ORA (high > 1.0 D; low ≤ 1.0 D). The baseline and 12-month postoperative data were analyzed. Data are expressed as mean ± standard deviation (SD). A value of *p* less than 0.05 was considered statistically significant.

**Results:**

For the TICL group, no significant differences in the postoperative RA, safety index, efficacy index, index of success (IOS), correction index (CI), and angle of error (AOE) were found between high (*n* = 36) and low ORA (*n* = 36) groups (Mann–Whitney *U* test, *p* > 0.05). For the SMILE group, the postoperative RA (high: −0.67 ± 0.43 D, low: −0.39 ± 0.29 D, Mann–Whitney *U* test, *p* = 0.003) and IOS (high: 0.50 ± 0.43, low: 0.25 ± 0.23, Mann–Whitney *U* test, *p* = 0.003) were greater in the high ORA group. When comparing TICL and SMILE groups, the mean postoperative RA (TICL: −0.48 ± 0.29 D, SMILE: −0.67 ± 0.43 D, Mann–Whitney *U* test, *p* = 0.03) and IOS (TICL: 0.32 ± 0.23, SMILE: 0.50 ± 0.43, Mann–Whitney *U* test, *p* = 0.03) were significantly higher in the SMILE group when the ORA was >1.0 D.

**Conclusion:**

Both TICL and SMILE are effective in correcting myopic astigmatism. ORA has a lesser effect on TICL than on SMILE.

## Introduction

Ocular astigmatism determined by manifest refraction is the sum of anterior corneal astigmatism and non-anterior corneal astigmatism (1). Astigmatism that cannot be attributed to the anterior corneal surface is referred to as ocular residual astigmatism (ORA; 2). Astigmatism can be corrected by glasses, contact lens, and surgeries. The two most widely accepted methods to correct astigmatism are laser correction and toric implantable collamer lens (TICL). Laser correction of astigmatism has been mainly based on subjective refractive astigmatism (RA). For eyes in which ORA is the main component, laser ablation might induce new astigmatism or result in excess remaining corneal astigmatism, potentially reducing the correction efficacy. Qian et al. have demonstrated that ORA influences the efficacy of laser-assisted *in situ* keratomileusis (LASIK), laser-assisted subepithelial keratomileusis (LASEK), and small incision lenticule extraction (SMILE) in correcting myopic astigmatism when the refractive correction is confined to the anterior cornea (3–5). In contrast to laser correction, TICL directly corrects astigmatism instead of ablating the cornea. Siedlecki et al. compared the effect of SMILE and TICL (V4c) on the correction of myopia or myopic astigmatism and found no difference in the postoperative mean refractive remaining cylinder between the two procedures (6).

In the current study, we compared the effect of ORA on the correction of TICL and SMILE for myopic astigmatism. To mitigate bias toward eyes with high RA in the low ORA group and eyes with low RA in the high ORA group, the preoperative RA and ORA were matched between the TICL and SMILE groups in this study.

## Patients and Methods

This prospective study included patients who underwent TICL implantation or SMILE for correction of myopia and myopic astigmatism from December 2018 to December 2019 at the Ophthalmology Department of the Eye and ENT Hospital, Shanghai, China. One-to-one matching between the TICL and SMILE groups was performed by preoperative manifest RA and ORA, with a maximum difference of 0.50 D for RA and 0.25 D for ORA. In each surgical group, patients were further divided according to ORA (high: >1D; low: ≤1D).

Exclusion criteria were the following: patients younger than 18 years or older than 45 years, the sum of sphere and astigmatism greater than −18.0 D for TILC or −15 D for SMILE, best corrected distance visual acuity less than 20/25, and other pathologic ocular conditions or relevant systemic diseases. This study followed the tenets of the Declaration of Helsinki and was approved by the ethics committee of the EENT Hospital of Fudan University. Informed written consent were obtained from all subjects.

### Small Incision Lenticule Extraction

All SMILE procedures were performed by an experienced surgeon (YS, Qian) using a VisuMax femtosecond laser system (Carl Zeiss Meditec AG, Jena, Germany) following the surgical procedure described by Sekundo with a repetition rate of 500 kHz and pulse energy of 130 nJ (7). Following the incision, the refractive lenticule was dissected and separated through the 2-mm side incision and manually removed. No intra-operative or postoperative complications were observed in all patients.

### ICL Implantation

Toric implantable collamer lens size was based primarily on white-to-white distance and anterior chamber depth measurements, as recommended by the STAAR surgical calculator^1^. Only patients with an anterior chamber depth of 2.8 mm or greater and a preoperative endothelial cell density of 2,000 cells/mm^2^ or greater were eligible for ICL implantation. All surgeries were performed by an experienced surgeon using the same technique (XT, Zhou). Standard TICL surgery was performed, and a temporal (180° for the right eye and 0° for the left eye) 3-mm corneal incision was made using the procedure described in our previous report. The mean flattening effect of the astigmatism induced by the incision was 0.51 D as demonstrated in a previous study (8). It was integrated into the preoperative planning for the TICL. Zero astigmatism was targeted in all eyes. Preoperative corneal marking of the desired axis was conducted by the surgeon at the slit lamp with two opposing marks placed with a marking pen.

### Measurements and Vector Analysis for Astigmatism

Patients were examined preoperatively and at 1, 3, 6, and 12 months postoperatively. The baseline and 12-month postoperative data were analyzed. Objective and subjective refraction tests were performed, and the logarithm of the minimum angle of resolution (logMAR) of uncorrected (UDVA) and CDVAs were recorded during all follow-up visits. Pentacam (Oculus GmbH, Wetzlar, Germany) imaging of the anterior surface was performed by an experienced examiner. Three measurements were averaged for each result. Manifest refraction started with objective refraction. The endpoint was the manifest refraction representing the total astigmatism of the eye and its perception. Postoperative rotational stability of the TICL was measured using OPD-Scan III (Nidek Co., Ltd.) as reported by Lee et al. (9). Eyes were excluded if the degree of rotation was ≥5°, which would lead to a visually significant change in the residual cylinder.

Topographic parameters included flat central radius (*R*_*f*_), steep central radius (*R*_*s*_), and meridian of the *R*_*f*_ on the central 3 mm ring of the anterior corneal surface. The power created by the anterior corneal surface in the flat meridian (*P*_*f*_) was $\frac{n_{c}-1}{{\text{R}}_{\text{f}}}$ and the power in the steep meridian (*P*_*s*_) was $\frac{n_{c}-1}{{\text{R}}_{\text{s}}}$, with *n*_*c*_ as the refractive index of the cornea (1.376; 10). Astigmatism of the anterior cornea in the central 3 mm ring in positive cylinder form is (*P*_*s*_ − *P*_*f*_), with the same axis as *P*_*s*_.

Ocular residual astigmatism was determined as the vector difference between preoperative manifest RA and astigmatism of the anterior cornea (2, 11). Preoperative manifest RA was converted to the corneal plane using a vertex of 12 mm. Surgical-induced astigmatism vector (SIA) is the amount and axis of astigmatic change caused by surgery. It was determined as the vector difference between the pre- and postoperative astigmatism determined by manifest refraction (2, 11).

The following indices were used for comparing: postoperative astigmatism determined by manifest refraction (or difference vector), surgical-induced astigmatism vector (SIA), index of success (IOS), correction index (CI), and angle of error (AOE). IOS is the ratio of postoperative manifest astigmatism to target-induced astigmatism vector (TIA). A higher IOS means a higher proportion of TIA uncorrected by surgery. CI is the ratio of SIA to TIA (the treatment applied by the laser or refractive implant power at the corneal plane). AOE is the angle between the axis of the SIA and the axis of the TIA. An AOE with a positive value refers to counterclockwise (cc/Wise) rotational error while an AOE with a negative value refers to clockwise (c/Wise) rotational error (10–12).

### Statistical Analysis

Statistical analyses were performed using SPSS software (13.0; SPSS Inc., Chicago, IL, United States). All data are reported as mean ± standard deviation (SD). For normally distributed parameters, independent samples *t*-tests were performed between high and low ORA groups, and paired *t*-tests were performed between TICL and SMILE groups. For non-normally distributed parameters, Mann–Whitney *U* tests were performed for two ORA groups and Wilcoxon signed-rank tests were performed for TICL and SMILE groups. The safety index was defined as the ratio of postoperative CDVA to preoperative CDVA. The efficacy index was defined as the ratio of postoperative UDVA to preoperative CDVA. A value of *p* less than 0.05 was considered statistically significant.

## Results

Two eyes with degrees of rotation ≥ 5° were excluded from the TICL group. Hence, a total of 72 eyes were implanted with TICL (high ORA: *n* = 36; low ORA: *n* = 36) and 72 eyes that underwent SMILE (high ORA: *n* = 36; low ORA: *n* = 36) for correction of myopia and myopic astigmatism were included. The baseline characteristics of the patients are summarized in Table 1.

**TABLE 1:** Comparisons between TICL and SMILE.

	TICL group			SMILE group		
	High ORA *n* = 36	Low ORA *n* = 36	*P* value	High ORA *n* = 36	Low ORA *n* = 36	*P* value
**Preop**						
Age (y)	28.36 ± 6.91 (18, 42)	28.03 ± 6.94 (18, 44)	0.760	27.75 ± 7.57 (19, 43)	27.17 ± 7.08 (18, 43)	0.838
Male/Female	8/28	15/21		24/12	12/24	
Sphere (D)	−8.05 ± 3.86 (−17.0, −2.5)	−9.01 ± 3.11 (−16.75, −3.25)	0.250	−4.49 ± 2.27 (−9.0, −1.25)	−6.21 ± 1.26 (−9.0, −3.5)	**0.001**
RA (D)	−1.78 ± 0.71 (−3.5, −0.5)	−2.03 ± 0.88 (−3.75, −1)	0.367	−1.64 ± 0.80 (−3.5, −0.25)	−1.87 ± 0.92 (−4.0, −0.75)	0.286
KA (D)	1.73 ± 0.84 (0.6, 3.3)	1.81 ± 0.76 (0.5, 3.8)	0.731	2.26 ± 0.76 (0.8, 4.1)	1.91 ± 0.82 (0.6, 3.9)	0.047
ORA (D)	1.32 ± 0.22 (1.01, 1.71)	0.54 ± 0.23 (0.16, 0.99)	**<0.001**	1.35 ± 0.21 (1.03, 1.73)	0.55 ± 0.27 (0.01, 0.95)	**<0.001**
CDVA (LOGMAR)	0.03 ± 0.08 (−0.08, 0.30)	0.04 ± 0.07 (−0.08, 0.30)	1.0	−0.01 ± 0.05 (−0.08, 0.10)	−0.01 ± 0.04 (−0.18, 0.05)	0.793
**Postop 12 m**						
UDVA (LOGMAR)	0.003 ± 0.10 (−0.08, 0.30)	0.04 ± 0.12 (−0.08, 0.30)	0.152	0.005 ± 0.12 (−0.18, 0.40)	−0.005 ± 0.07 (−0.18, 0.22)	0.577
CDVA (LOGMAR)	−0.05 ± 0.05 (−0.18, 0.05)	−0.02 ± 0.07 (−0.08, 0.15)	0.211	−0.03 ± 0.05 (−0.18, 0)	−0.02 ± 0.05 (−0.18, 0.10)	0.318
Degree of rotation (°)	2.17 ± 1.11 (0, 4)	2.47 ± 1.23 (0, 4)	0.293	NA	NA	NA
Safety index	1.21 ± 0.19 (1.00, 1.80)	1.15 ± 0.12 (1.00, 1.50)	0.244	1.06 ± 0.12 (0.83, 1.25)	1.04 ± 0.16 (0.80, 1.50)	0.305
Efficacy index	1.09 ± 0.21 (0.56, 1.50)	1.01 ± 0.20 (0.60, 1.33)	0.090	0.99 ± 0.20 (0.44, 1.25)	1.01 ± 0.19 (0.60, 1.50)	0.990
Sphere (D)	0.04 ± 0.62 (−1.0, 1.0)	−0.31 ± 0.56 (−1.5, 1.0)	**0.005**	−0.17 ± 0.74 (−1.0, 1.0)	0.01 ± 0.63 (−1.25, 1.0)	0.330
Cylinder (D)	−0.48 ± 0.29 (−1.0, 0)	−0.42 ± 0.27 (−0.75, 0)	0.492	−0.67 ± 0.43 (−1.75, 0)	−0.39 ± 0.29 (−1.0, 0)	**0.003**
IOS	0.32 ± 0.23 (0, 1.01)	0.27 ± 0.28 (0, 1.51)	0.236	0.50 ± 0.43 (0, 1.68)	0.25 ± 0.23 (0, 1.01)	**0.003**
CI	0.95 ± 0.32 (0.17, 1.5)	0.91 ± 0.17 (0.51, 1.5)	0.305	0.91 ± 0.45 (0.14, 2.65)	0.99 ± 0.22 (0.69, 1.75)	0.061
AOE (Arith. mean)	2.39 ± 10.11 (−16.8, 40.0)	0.29 ± 8.39 (−20.6, 23.6)	0.445	2.87 ± 19.38 (−32.5, 62.9)	1.24 ± 7.38 (−14.8, 25.0)	0.973
AOE (Abs. mean)	5.8 ± 1.4 (0, 40)	6.0 ± 5.8 (0, 23.6)	0.357	10.9 ± 16.2 (0, 62.9)	4.8 ± 5.7 (0, 25)	0.076

*All values are reported as mean ± SD (range). RA, refractive astigmatism; ORA, ocular residual astigmatism; CDVA, corrected distance visual acuity; UDVA, uncorrected distance visual acuity; IOS, index of success; CI, correction index; SIA/TIA, surgical-induced astigmatism vector/target-induced astigmatism vector; AOE, angle of error; Arith. Mean, arithmetic mean; Abs. mean, absolute value mean; and NA, not applicable. A value of p less than 0.05 was considered statistically significant.*

### Comparisons Between High and Low Ocular Residual Astigmatism Groups

Table 1 lists the results of several indices comparing the high and low ORA groups. For the TICL group, no significant differences in preoperative spherical error (independent-samples *t*-test, *t* = 0.16, *p* = 0.25) and RA (Mann–Whitney *U* test, *p* = 0.367) were found between the high and low ORA groups. The postoperative rotation of TICL was 2.17 ± 1.11° for the high ORA group and 2.47 ± 1.23° for the low ORA group (Mann–Whitney *U* test, *p* = 0.293). Postoperatively, no significant differences in the RA, UDVA (logMAR), CDVA (logMAR), safety index, efficacy index, IOS, CI, and AOE were found between the high and low ORA groups (Mann–Whitney *U* test, *p* > 0.05). The postoperative spherical error was greater in the low ORA group (high ORA group: 0.04 ± 0.62 D, low ORA group: −0.31 ± 0.56 D, Mann–Whitney *U* test, *p* = 0.005).

For the SMILE group, the preoperative spherical error was higher in the low ORA group (high ORA group: −4.49 ± 2.27 D, low ORA group: −6.21 ± 1.26 D, *t* = 3.97, *p* < 0.001). No significant differences were found in the preoperative RA between the high and low ORA groups (Mann–Whitney *U* test, *p* = 0.286). Both the postoperative RA (high ORA group: −0.67 ± 0.43 D, low ORA group: −0.39 ± 0.29 D, Mann–Whitney *U* test, *p* = 0.003) and IOS (high ORA group: 0.50 ± 0.43, low ORA group: 0.25 ± 0.23, Mann–Whitney *U* test, *p* = 0.003) were higher in the high ORA group. No significant differences in the postoperative UDVA (logMAR), postoperative CDVA (logMAR), postoperative spherical error, safety index, efficacy index, CI, and AOE were found between the high and low ORA groups (Mann–Whitney *U* test, *p* > 0.05).

### Comparisons Between Toric Implantable Collamer Lens and Small Incision Lenticule Extraction Groups

**FIGURE 1:**
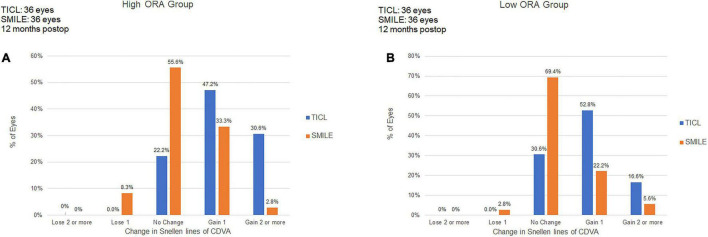
Procedural safety for small incision lenticule extraction (SMILE) and toric implantable Collamer lens (TICL) implantation (Visian Implantable Collamer Lens; STAAR Surgical, Monrovia, CA, United States) in the high ORA **(A)** and low ORA **(B)** groups.

For the high ORA group, 8.3% of eyes in the SMILE group lost one line, whereas no eye in the TICL group lost any CDVA. No eyes lost two or more lines of CDVA (Figure 1). The safety index was higher in the TICL (1.21 ± 0.19) than in the SMILE (1.06 ± 0.12) group, as it was assessed using the Wilcoxon signed-rank test (*p* < 0.001). For the low ORA group, 2.8% of eyes in the SMILE group lost one line, whereas no eye in the TICL group lost any CDVA. The safety index was higher in the TICL (1.15 ± 0.12) than in the SMILE (1.04 ± 0.16) group (Wilcoxon signed-rank test, *p* = 0.004).

**FIGURE 2:**
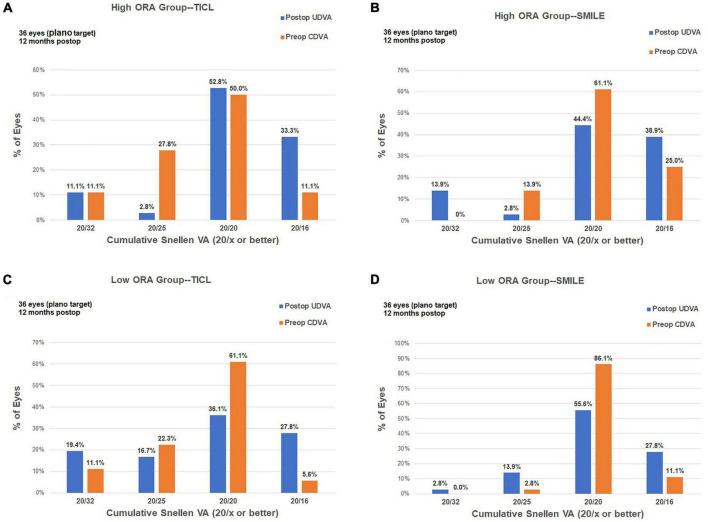
Procedural efficacy for small incision lenticule extraction (SMILE) and toric implantable Collamer lens (TICL) implantation (Visian Implantable Collamer Lens; STAAR Surgical, Monrovia, CA, United States) in the high ORA **(A,B)** and low ORA **(C,D)** groups.

For the high ORA group, the efficacy index was significantly higher in the TICL (1.09 ± 0.21) than in the SMILE (0.99 ± 0.20) group (Wilcoxon signed-rank test, *p* = 0.017). For the low ORA group, no significant difference was found in the efficacy index between TICL (1.01 ± 0.20) and SMILE (1.01 ± 0.19) groups (Wilcoxon signed-rank test, *p* = 0.887, Figure 2).

**FIGURE 3:**
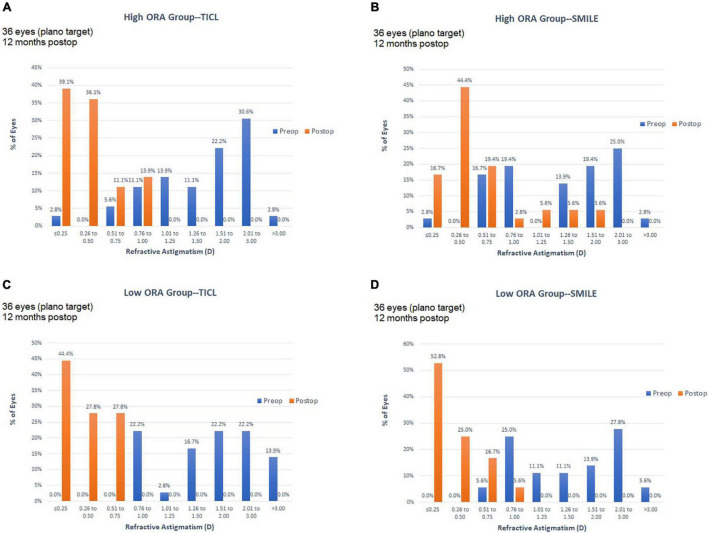
Refractive astigmatic accuracy for small incision lenticule extraction (SMILE) and toric implantable Collamer lens (TICL) implantation (Visian Implantable Collamer Lens; STAAR Surgical, Monrovia, CA, United States) in the high ORA **(A,B)** and low ORA **(C,D)** groups.

**FIGURE 4:**
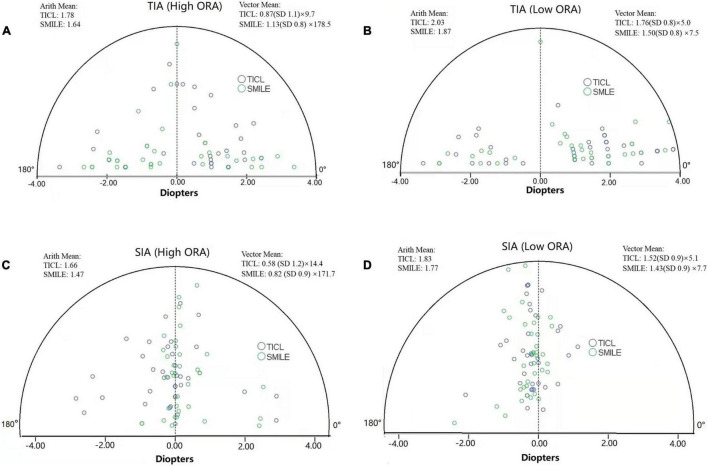
Target-induced astigmatism vector (TIA, **A,B**) and surgically induced astigmatism vector (SIA, **C,D**) in high and low ORA groups on polar diagrams for toric implantable Collamer lens (TICL) implantation (Visian Implantable Collamer Lens; STAAR Surgical, Monrovia, CA, United States) and small incision lenticule extraction (SMILE) for the high and low ORA groups (Arith., arithmetic; ORA, ocular residual astigmatism).

**FIGURE 5:**
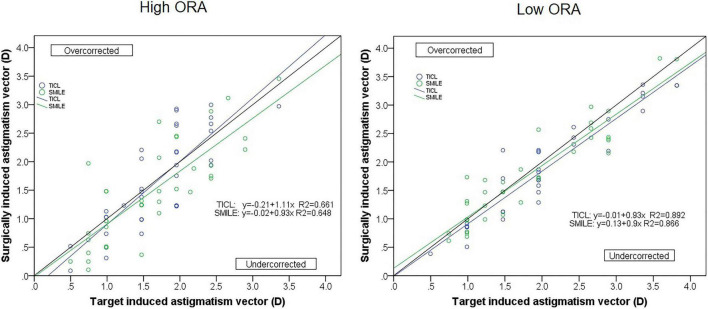
Target-induced astigmatism vector (TIA) plotted against surgical-induced astigmatism vector (SIA) for small incision lenticule extraction (SMILE) and toric implantable Collamer lens (TICL) implantation (Visian Implantable Collamer Lens; STAAR Surgical, Monrovia, CA, United States) for the high and low ORA groups.

**FIGURE 6:**
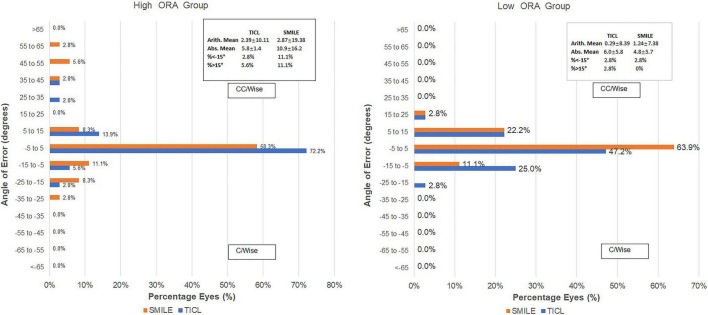
Distribution of angle of error (AOE) after small incision lenticule extraction (SMILE) and toric implantable Collamer lens (TICL) implantation (Visian Implantable Collamer Lens; STAAR Surgical, Monrovia, CA, United States) for the high and low ORA groups.

For the high ORA group, both the postoperative manifest RA (TICL: −0.48 ± 0.29 D, SMILE: −0.67 ± 0.43 D, Wilcoxon signed-rank test, *p* = 0.033, Figure 3) and IOS (TICL: 0.32 ± 0.23, SMILE: 0.50 ± 0.43, Wilcoxon signed-rank test, *p* = 0.035) were significantly higher in the SMILE than in the TICL group. No significant difference was found in the postoperative sphere, CI, or AOE between the two groups. For the low ORA group, no significant differences in the postoperative sphere, RA (Figure 3), IOS, CI, or AOE were found between the SMILE and the TICL groups (*p* > 0.05). Figure 4 shows the TIA and SIA in high and low ORA groups on polar diagrams. Figure 5 shows the SIA plotted against TIA in high and low ORA groups. Figure 6 shows the distributions of AOE for both groups.

## Discussion

In this study, we found that for eyes that underwent SMILE, the postoperative RA, and IOS were greater in the high ORA group than in the low ORA group. Meanwhile, no significant difference in postoperative RA and IOS was found between the high and low ORA groups for TICL eyes. When comparing between TICL and SMILE groups, the postoperative RA and IOS were greater in eyes that underwent SMILE than in eyes implanted with TICL in the high ORA group. Meanwhile, no significant differences were found between the two procedures for the low ORA group. We infer from these results that ORA may influence the correcting efficiency of laser refractive surgery only. TICL showed higher predictability of surgical success in the correction of astigmatism in eyes with ORA > 1 D.

Several studies have confirmed the influence of ORA on the correction efficacy of laser-refractive surgeries, such as LASIK (3, 13, 14), LASEK (4), and SMILE (5, 15, 16). Sculpting the cornea based only on manifest refraction has the disadvantage that the entire ORA remains on the cornea as the postoperative surgical residual astigmatism, resulting in induction of higher order aberrations in some cases. Moreover, changes in the partial compensation between anterior corneal astigmatism and internal astigmatism could be a cause of reduced predictability in eyes with high ORA. In patients with high TIA (>0.5 D) and high ORA, Alpins suggested vector planning with both topographic and refractive values prior to surgical procedure (17, 18). Treatment would leave reduced residual astigmatism on the cornea (instead of the customary 100%) and a theoretical portion of residual astigmatism in RA (instead of the customary 0%). Arbelaez et al. showed that the vector planning strategy resulted in comparable visual and refractive outcomes and better postoperative corneal toricity as compared to conventional manifest refraction-based treatment in a group of patients who underwent LASIK procedures (14). The vector planning method was also applied in SMILE and yielded improved outcomes for all three astigmatism parameters of refractive, corneal, and ORA in eyes with high ORA (15, 16).

In contrast to laser-refractive surgery, TICL implantation removes the need of sculpting the cornea. To our knowledge, no study has been reported on the impact of ORA on the correction of myopic astigmatism by TICL. In this study, we found no significant difference in postoperative RA and IOS between high and low ORA groups for TICL eyes. Therefore, TICL should be recommended for eyes with high ORA. However, several factors should still be taken into consideration. First, the flattening effect and mean magnitude SIA should be included in the preoperative planning if a clear corneal incision was made for TICL implantation. Second, the cylinder correction effect is decreased if the lens rotates after surgery. It is well accepted that a rotation of the intraocular lens 10° away from the intended implantation axis increases refraction and decreases optical performance, and a rotation of 30° has no effect on correcting prevailing astigmatism so magnitude does not change but the axis of astigmatism rotates by 30°. A TICL with a larger size can be selected to increase stability if the depth of the anterior chamber is sufficient (19, 20).

With respect to correction efficacy of astigmatism, we found no significant difference between the two procedures in eyes with low ORA. Siedlecki also reported that there was no difference in postoperative mean residual cylinder in the ±0.50 and ±1.00 D astigmatic accuracy between TICL and SMILE (6). Several studies have also found no significant differences in the safety or efficacy index between these two procedures (21–23). In contrast, Moshirfar et al. reported that SMILE was more accurate than TICL within ±0.25, ±0.50, and ±1.00 D of cylinder (24). Wan et al. also found that the accuracy of correction in the magnitude and axis of astigmatism was better in SMILE than in TICL regardless of the magnitude of TIA (20). They attributed the discrepancy between the two procedures to the greater increments for astigmatism for TICL (0.50 D vs. 0.01 D), the corneal astigmatic changes induced by the 3-mm corneal incision for TICL, “non-zero” target prevailing in TICL, and postoperative rotation for TICL.

## Limitations of the Study

In this study, the preoperative RA was matched between the TICL and SMILE groups whereas the preoperative spherical error was much higher in the TICL group. Additionally, no marking and cyclotorsion compensation were used for the SMILE group, which can lead to less correction of astigmatism. These factors may lead to bias in the comparison between TICL and SMILE. However, the comparison between high and low ORA groups for the same procedure would not be biased. Finally, only visual acuity was assessed in this study, while other visual parameters, such as halos and contrast sensitivity, which are possibly correlated with postoperative astigmatism, (22) were not evaluated. Further study with more stringent matching and with cyclotorsion compensation for both procedures is warranted to further evaluate the advantages and drawbacks of SMILE and ICL implantation.

## Conclusion

Both TICL and SMILE are effective in correcting myopic astigmatism. However, SMILE may be less effective when the astigmatism is mainly ORA. ORA should be considered in surgical planning for SMILE instead of considering manifest astigmatism alone. For TICL, no significant difference was found in the correction of astigmatism between eyes with high and low ORA. Therefore, TICL should be recommended for eyes with high ORA.

## Data Availability Statement

The original contributions presented in this study are included in the article/supplementary material, further inquiries can be directed to the corresponding authors.

## Ethics Statement

The studies involving human participants were reviewed and approved by the Ethics Committee of the EENT Hospital of Fudan University. The patients/participants provided their written informed consent to participate in this study.

## Author Contributions

LS: conceptualization, data collection, and manuscript drafting and critical revision. XYZ: data collection and manuscript drafting. LD and YS: data collection and analyzing. YQ and XTZ: conceptualization, critical revision of manuscript, and funding and supervision. All authors approved the final submission of this manuscript.

## Conflict of Interest

The authors declare that the research was conducted in the absence of any commercial or financial relationships that could be construed as a potential conflict of interest.

## Publisher’s Note

All claims expressed in this article are solely those of the authors and do not necessarily represent those of their affiliated organizations, or those of the publisher, the editors and the reviewers. Any product that may be evaluated in this article, or claim that may be made by its manufacturer, is not guaranteed or endorsed by the publisher.

## Footnotes

1
http://www.staarvision.com
